# Unusual hydrocephalus from retrocerebellar arachnoid cyst sparing the fourth ventricle

**DOI:** 10.11604/pamj.2025.51.30.45626

**Published:** 2025-06-04

**Authors:** Devyansh Nimodia, Sakshi Dudhe

**Affiliations:** 1 Department of Radiodiagnosis, Datta Meghe Institute of Medical Sciences, Sawangi, Wardha, Maharashtra, India

**Keywords:** Quadrigeminal cistern, hydrocephalus, retrocerebellar cysts

## Image in medicine

A 17-year-old male presented to our medicine outpatient department with complaints of worsening headache, nausea and intermittent vomiting for the past two months. He had similar complaints in the past, which were relieved by taking. The patient was advised to do a computed tomography scan of the brain, which revealed a cerebrospinal fluid (CSF) density cystic lesion measuring approximately.8.5 x 6.7 x 8.2 cm noted in the retro cerebellar region extending to the quadrigeminal cistern, causing moderate compression of bilateral cerebellar hemispheres and cerebral aqueduct leading to moderate dilatation of bilateral lateral ventricles, 3^rd^ ventricle (A). Notably, the fourth ventricle remained of normal size (B), which is a rare occurrence in such cases where obstruction of CSF flow typically affects the fourth ventricle. The patient was operated on by transventricular approach with endoscopic third ventriculocystostomy and endoscopic third ventriculostomy (ETV). The postoperative course was uneventful. The patient was further advised to do MRI CSF flowmetry, which was not done for financial reasons. Retrocerebellar cysts are rare and can sometimes obstruct CSF pathways, leading to hydrocephalus. This case is unique because the fourth ventricle remained unaffected despite significant hydrocephalus involving the lateral and third ventricles. One possible explanation is that the cyst’s primary impact was at the level of the quadrigeminal cistern and aqueduct of Sylvius, leading to an obstructed outflow of CSF from the third ventricle, while the CSF circulation through the fourth ventricle remained relatively unimpeded. This case also emphasizes the importance of early surgical intervention.

**Figure 1:**
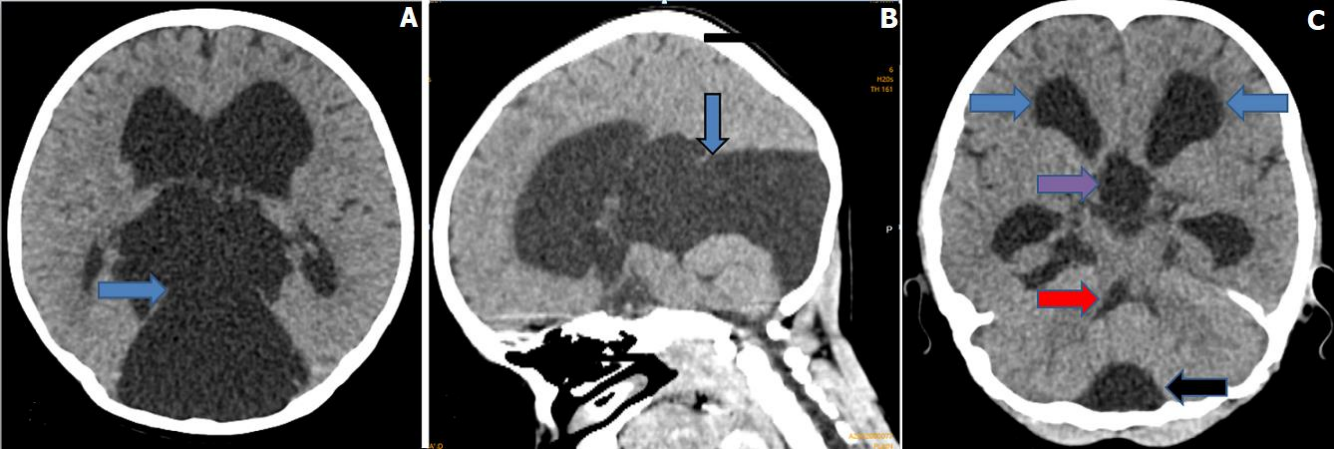
computed tomography scan axial section (A) and sagittal section (B) brain window showing retrocerebellar cyst communicating with quadrigeminal cistern with dilatation of bilateral lateral ventricle and third ventricle; (C) computed tomography scan axial section brain window showing retrocerebellar cyst (black arrow), dilatated bilateral lateral ventricle (blue arrow) and third ventricle (purple arrow), note the non-dilated fourth ventricle (red arrow)

